# Racial inequalities in child vaccination and barriers to vaccination in Brazil among live births in 2017 and 2018: an analysis of a retrospective cohort of the first two years of life

**DOI:** 10.1590/S2237-96222024v33e20231216.especial2.en

**Published:** 2024-08-23

**Authors:** Antonio Fernando Boing, Alexandra Crispim Boing, Ana Paula França, José Cássio de Moraes, ICV 2020 Group, Adriana Ilha da Silva, Alberto Novaes Ramos, Ana Paula França, Andrea de Nazaré Marvão Oliveira, Antonio Fernando Boing, Carla Magda Allan Santos Domingues, Consuelo Silva de Oliveira, Ethel Leonor Noia Maciel, Ione Aquemi Guibu, Isabelle Ribeiro Barbosa Mirabal, Jaqueline Caracas Barbosa, Jaqueline Costa Lima, José Cássio de Moraes, Karin Regina Luhm, Karlla Antonieta Amorim Caetano, Luisa Helena de Oliveira Lima, Maria Bernadete de Cerqueira Antunes, Maria da Gloria Teixeira, Maria Denise de Castro Teixeira, Maria Fernanda de Sousa Oliveira Borges, Rejane Christine de Sousa Queiroz, Ricardo Queiroz Gurgel, Rita Barradas Barata, Roberta Nogueira Calandrini de Azevedo, Sandra Maria do Valle Leone de Oliveira, Sheila Araújo Teles, Silvana Granado Nogueira da Gama, Sotero Serrate Mengue, Taynãna César Simões, Valdir Nascimento, Wildo Navegantes de Araújo

**Affiliations:** 1 Universidade Federal de Santa Catarina, Programa de Pós-graduação em Saúde Coletiva, Florianópolis, SC, Brazil; 2 Faculdade de Ciências Médicas Santa Casa de São Paulo, Departamento de Saúde Coletiva, São Paulo, SP, Brasil; 3 Universidade Federal do Espírito Santo, Vitória, ES, Brazil; 4 Universidade Federal do Ceará, Departamento de Saúde Comunitária, Fortaleza, CE, Brazil; 5 Faculdade Ciências Médicas Santa Casa de São Paulo, São Paulo, SP, Brazil; 6 Secretaria de Estado da Saúde do Amapá, Macapá, AP, Brazil; 7 Universidade Federal de Santa Catarina, SC, Brazil; 8 Organização Pan-Americana da Saúde, Brasília, DF, Brazil; 9 Instituto Evandro Chagas, Belém, PA, Brazil; 10 Faculdade de Ciências Médicas Santa Casa de São Paulo, Departamento de Saúde Coletiva, São Paulo, SP, Brazil; 11; 12; 13 Universidade Federal de Mato Grosso, Cuiabá, MT, Brazil; 14 Universidade Federal do Paraná, Curitiba, PR, Brazil; 15 Universidade Federal de Goiás, Goiânia, GO, Brazil; 16 Universidade Federal do Piauí, Teresina, PI, Brazil; 17 Universidade de Pernambuco, Faculdade de Ciências Médicas, Pernambuco, PE, Brazil; 18 Instituto de Saúde Coletiva, Universidade Federal da Bahia, Salvador, BA, Brazil; 19 Secretaria de Estado da Saúde de Alagoas, Maceió, AL, Brazil; 20 Universidade Federal do Acre, Rio Branco, AC, Brazil; 21 Universidade Federal do Maranhão, Departamento de Saúde Pública, São Luís, MA, Brazil; 22 Universidade Federal de Sergipe, Aracaju, SE, Brazil; 23 Secretaria Municipal de Saúde, Boa Vista, RR, Brazil; 24 Fundação Oswaldo Cruz, Mato Grosso do Sul, Campo Grande, MS, Brazil; 25 Fundação Oswaldo Cruz, Escola Nacional de Saúde Pública Sergio Arouca, Rio de Janeiro, RJ, Brazil; 26 Universidade Federal do Rio Grande do Sul, Porto Alegre, RS, Brazil; 27 Fundação Oswaldo Cruz, Instituto de Pesquisa René Rachou, Belo Horizonte, MG, Brazil; 28 Secretaria de Desenvolvimento Ambiental de Rondônia, Porto Velho, RO, Brazil; 29 Universidade de Brasília, Brasília, DF, Brazil

**Keywords:** Cobertura de Vacunación, Niño, Inequidades en Salud, Encuestas Epidemiológicas, Brasil, Vaccination Coverage, Child, Health Inequities, Health Surveys, Brazil

## Abstract

**Objective:**

To describe timely vaccination completion and obstacles in the first 24 months of life in Brazil, examining associations with maternal race/skin color.

**Methods:**

Study participants were 37,801 children born in 2017 and 2018 included in the National Immunization Coverage Survey. We calculated prevalence and 95% confidence intervals for timely vaccine completeness and obstacles at 5, 12 and 24 months of life, according to maternal race/skin color. Associations were analyzed using logistic regression.

**Results:**

7.2% (95%CI 6.3;8.2) of mothers faced difficulties in taking their children to be vaccinated, and 23.4% (95%CI 21.7;25.1) were not vaccinated when taken. These proportions were 75% (95%CI 1.25;2.45) and 97% (95%CI 1.57;2.48) higher, respectively, among Black mothers. At least one vaccination was delayed among 49.9% (95%CI 47.8;51.9) and 61.1% (95%CI 59.2;63.0) of children by 5 and 12 months, respectively. These rates were higher among Black/mixed race mothers.

**Conclusion:**

There are racial inequalities in both the obstacles faced and in vaccination rates in Brazil.

## Study contributions

**Main results:**

Marked racial inequalities were found in the obstacles to vaccination of children under 24 months in Brazil and to timely vaccination at 5 months and in the first year of life.

**Implications for services:**

Racial inequalities in the occurrence of vaccination shortcomings in health services, in the objective restrictions faced by families in taking their children to vaccination centers and in incomplete vaccination in a timely manner need to be addressed by the Brazilian National Health System.

**Perspectives:**

Equal public policies to address barriers to vaccination and qualification of health services need to be implemented. Studies need to deepen understanding of the structural determinants that lead to racial disparities.

## RESUMEN

**Objetivo:**

Describir completitud vacunal a tiempo en los primeros 24 meses de vida en Brasil y obstáculos para vacunar, evaluando asociaciones con etnicidad materna.

**Métodos:**

Análisis de datos de nacidos en 2017 y 2018, participantes de la Encuesta Nacional de Cobertura Vacunal a lo largo de sus primeros 24 meses de vida. Verificación de prevalencia y los intervalos de confianza 95% de obstáculos vacunales y completitud a los cinco meses, primer y segundo año por color/raza materna. Se utilizó regresión logística para el análisis de asociaciones.

**Resultados:**

Datos de 37.801 niños analizados. El 7,2% (IC_95_% 6,3;8,2) enfrentó dificultades para vacunarse y el 23,4% (IC_95_% 21,7; 25,1) no se vacunó incluso llevados. Estas cifras fueron 75% (IC_95_% 1,25;2,45) y 97% (IC_95_% 1,57;2,48) más altas entre madres negras. El 49,9% (IC_95_% 47,8;51,9) y 61,1% (IC_95_% 59,2;63,0) experimentaron retrasos vacunales a los cinco meses y al año, respectivamente, con tasas superiores en pardos/negros.

**Conclusión:**

Desigualdades raciales en obstáculos y vacunación en Brasil.

## INTRODUCTION

Immunization plays a fundamental role in preventing infectious diseases and reducing child mortality, but its effective distribution is uneven both between countries and within them.^1^ The World Health Organization (WHO) estimates that in the 2010s more than 20 million babies did not complete the basic vaccination schedule around the world each year and, of these, more than 13 million did not receive any vaccine through immunization programs.^2^

The WHO has also set as one of its objectives provision of accessible vaccination services to all people everywhere by 2030, with the aim of saving more than 50 million lives.^2^ However, one of the crucial challenges facing this is overcoming existing disparities in countries. Among the multiple inequalities that challenge full human development are those related to ethnic-racial characteristics. Therefore, research that sheds light on them is essential for improving public policies aimed at equality,^3^ with racial disparities in childhood vaccination being influenced by skin color and a myriad of social, economic and health factors. 

Research covering 64 low- and middle-income countries found significant disparities in childhood immunization related to ethnicity in more than half of those countries.^4^ Several studies indicate that Black people face significant barriers in accessing vaccines, leading to a lower immunization rate when compared to other racial groups.^5,6^ Centers for Disease Control and Prevention (CDC)analysis indicates that in the United States, vaccine coverage against COVID-19 was lower among African-American children aged 5 to 11.^7^ Other studies also show poorer coverage among Black people and/or minority groups^8^ both in terms of vaccination and intention to vaccinate.^9^

Such inequality can result in higher rates of vaccine-preventable diseases and contribute to perpetuating health disparities.^1,2^ Brazil is marked by profound racial, social and economic inequalities with profound impacts on the health levels of its population. Therefore, expanding knowledge about race/skin color differences in access to vaccination and timely vaccination completion among children is necessary to evaluate and qualify public policies, especially in the face of a health system based on the principle of equity.

The objective of this study was to describe the magnitude of timely vaccination completion throughout the first 24 months of life in Brazil and obstacles to vaccination, testing their associations with maternal race/skin color.

## METHODS

### Data source

We analyzed data from the National Immunization Coverage Survey, a retrospective cohort carried out in Brazil between September 2020 and March 2022. All children born in 2017 and 2018 were included in the study and data were collected on vaccines received in the first 24 months of life. Analysis of the children’s vaccination cards enabled verification of the evolution of their exposure to recommended vaccines at each age during their first two years of life. Vaccination coverage in the fifth, twelfth and twenty-fourth months of life was analyzed. The survey was carried out in all 26 Brazilian state capitals of Brazil, its Federal District and also in 12 other cities with more than 100,000 inhabitants distributed throughout all regions of Brazil, with the exception of the Northern region, in non-metropolitan areas.

### Sample size calculation

The parameters used to calculate the sample size were estimated vaccination coverage prevalence of 70%, estimation error equal to 5%, 95% confidence interval, and design effect due to the use of clusters equal to 1.4. This resulted in a sample of 452 children per survey. Between one and four surveys were carried out in each municipality, depending on the number of live births in 2017 and 2018, totaling 89 surveys. The municipalities were taken to have four socioeconomic strata, according to census tract income and education characteristics. Further details of the sampling procedure can be found in a previous publication.^10^

### Data collection

The children’s addresses were obtained from the Live Birth Information System, which brings together information on all births occurring in Brazil. A closed structured questionnaire was administered to the interviewees using an electronic device with questions about the socioeconomic profile of the household and the sociodemographic profile of the mother and the person responsible for the child, if that person was not the mother. Information was also requested on perceptions regarding vaccines and barriers to vaccination. Additionally, the child’s vaccination card was requested for photographic recording. All data found in the photographs were typed by health professionals with knowledge of the national immunization schedule in order to record the vaccines administered and their respective administration dates. The study’s national coordinating body carried out data consistency analysis.

### Outcomes

Initially, we analyzed two outcomes related to objective difficulties in vaccinating children. Firstly, the respondent was asked whether they had ever had difficulty in taking their child to the vaccination center. Secondly, they were asked whether their child had failed to be vaccinated, even though they had been taken to the vaccination center. The answer options in both cases were “yes”, “no” or “doesn’t know/doesn’t want to say”. In the case of people who answered the first question affirmatively, they were asked whether the difficulty occurred because (1) they did not have the vaccination card, (2) they did not have time to take the child to be vaccinated, (3) the opening times of the vaccination center were inadequate, (4) the vaccination center was distant from their home or work, (5) their boss did not allow them time off to go to the vaccination center, (6) there were no means of transport for getting to the vaccination center, (7) they had no money to pay for getting to the vaccination center, (8) the person responsible for the child had a physical disability or health problem that hindered their mobility, (9) they did not know when the child should be vaccinated, (10) the child was sick, (11) other reason.

Similarly, those who reported that their child had not been vaccinated even though they had taken it to the vaccination center were asked what the reasons were: (1) no vaccine, (2) no supplies for administering the vaccine, (3) no medical professional in the vaccination room, (4) no more line number tickets available, (5) the vaccine room closed, (6) it was not the right day for the vaccination in question, (7) there were a lot of people in the line and they could not wait, (8) they did not vaccinate the child due to lack of documents (e.g. proof of address, Brazilian National Health System (SUS) card, or vaccination card), (9) the health professional advised against administering several vaccines on the same day and asked them to come back another day, (10) other reason. The interviewed person could answer “yes”, “no”, or “doesn’t know/doesn’t want to say” to each of these questions.

Three outcomes were analyzed regarding vaccination. Each considered a time frame over the first two years of the child’s life:

Delay or lack of access to vaccines that should be taken on time in the first five months of life: delay in bacillus Calmette-Guérin (BCG) and/or hepatitis B and/or 5-in-1 vaccine (diphtheria, tetanus, pertussis, hepatitis B and *Haemophilus influenzae* type B) (1^st^ or 2^nd^ dose) and/or inactivated poliovirus vaccine (IPV) (1^st^ or 2^nd^ dose) and/or rotavirus vaccine (1^st^ or 2^nd^ dose) and/or pneumococcal vaccine (1^st^ or 2^nd^ dose) and/or meningitis C vaccine (1^st^ or 2^nd^ dose).

Vaccine schedule not completed on time at 1^st^ year of life: BCG, hepatitis B, three doses of 5-in-1 vaccine and IPV, two doses of rotavirus vaccine, two doses of meningitis C vaccine and pneumococcal vaccine. 

Vaccine schedule not completed on time at 2^nd^ year of life: two doses of MMR (measles, mumps and rubella), one dose of hepatitis A, chickenpox and attenuated oral poliovirus vaccine (OPV); and a booster dose of DPT vaccine (diphtheria, pertussis and tetanus), meningitis C and pneumococcal vaccine.

The definition of whether or not the doses were timely and whether or not there was a delay was based on the time when they were administered, taking the date of birth and considering the interval between doses. The systematization of the intervals considered for each vaccine and dose can be found in Barata et al.^10^

### Covariables

The main variable of interest was the self-reported race/skin color of the children’s mothers. The answer options followed those used by the Brazilian Institute of Geography and Statistics (IBGE) in surveys conducted in Brazil: White, mixed race, Black, Indigenous and Asian. People who reported Indigenous or Asian race/skin color were not analyzed in the present study due to the small number found. Additionally, the following were included in the study as adjustment variables: maternal schooling (up to 8 years of study, 9 to 12 and 13 years or more), maternal work or internship for at least one hour per week in the last month doing an activity paid for with money (yes or no), and maternal age in years (up to 21, 22-29, 30-39 and 40 or over).

### Data analysis

Initially, the composition of the sample was described according to the covariables and the prevalence rates were estimated, with respective 95% confidence intervals (95%CI), for the five outcomes for the entire sample and according to each category of variables included in the study. We then calculated the relative frequencies of each of the 11 difficulties included in the study in taking the child to the vaccination center and each of the ten explanatory reasons for not vaccinating the child even when having taken it to the vaccination center. Finally, logistic regression was used to estimate crude and adjusted odds ratios, with respective 95%CI, between outcomes and covariables. All analyses considered the sample weights and the study design, and were performed using Stata 15.1. The National Immunization Coverage Survey was approved by the Human Research Ethics Committee of the Irmandade da Santa Casa de São Paulo under opinion No. 4.380.019.

## RESULTS

We analyzed data on 37,801 children. Losses accounted for 6%. Most respondents reported mixed race/skin color (46.4%), had more than 12 years of study (76.1%), were in the 30-39 year age group (46.5%) and had worked in the month before the interview (55.5%). We found that 7.2% had faced difficulties taking their children to the vaccination center and 23.4% of children were not vaccinated even after being taken to the center (Table 1). Furthermore, 49.9% of children experienced delays in receiving one or more vaccines on the schedule by the time they were 5 months old. In the first and second year of life, timely vaccination was incomplete for 61.1% and 86.1% of children, respectively. We found that the prevalence of difficulties in vaccinating, delay in vaccination and incomplete vaccination on time in the first year of life were higher among mothers of Black and mixed race/skin color compared to White mothers. In general, poorer indicators were also found among mothers with less education and those under 21 years old.

**Table 1:** Maternal characteristics of the sample and prevalence of outcomes analyzed in relation to timely vaccination coverage and difficulties in vaccinating children born between 2017 and 2018 in Brazil

	Sample	Faced difficulty in taking child to vaccination center	Child not vaccinated, despite having been taken to vaccination center	Delay in scheduled vaccination at up to 5 months old	Vaccine schedule not completed on time at 1st year of life	Vaccine schedule not completed on time at 2nd year of life
	**N**	**% (95%CI)**				
**All**	**37,801**	**7.2 (6.3;8.2)**	**23.4 (21.7;25.1)**	**49.9 (47.8;51.9)**	**61.1 (59.2;63.0)**	**86.1 (84.9;87.2)**
**Mother’s race/skin color (n = 36,341)**						
White	15,227	5.1 (4.1;6.5)	17.3 (15.5;19.2)	43.6 (40.8;46.4)	54.6 (51.8;57.4)	86.5 (84.5;88.4)
Mixed race	16,859	8.6 (7.5;9.8)	28.9 (26.8;31.2)	53.2 (48.2;58.2)	66.2 (63.9;68.4)	86.2 (84.6;87.6)
Black	4,255	8.7 (6.9;10.9)	29.2 (25.2;33.5)	54.9 (52.5;57.5)	65.4 (60.9;69.7)	83.1 (79.8;85.9)
**Mother’s schooling ( years of study) (n = 37,748)**						
Up to 8	3,280	11.2 (8.6;14.3)	23.2 (19.5;27.4)	68.0 (62.8;72.8)	75.4 (70.2;80.0)	85.3 (81.3;88.6)
9-12	5,494	10.2 (7.6;13.7)	23.8 (20.2;27.8)	55.8 (51.0;60.5)	64.3 (59.5;68.8)	83.8 (79.7;87.2)
13 or over	27,974	6.1 (5.3;7.0)	25.6 (23.4;29.9)	49.5 (46.6;52.3)	60.7 (58.1;63.2)	84.1 (82.3;85.7)
**Mother worked in the last month (n** **= 36,866)**						
No	16,420	8.0 (6.7;9.7)	25.3 (23.0;27.7)	51.7 (49.1;54.3)	61.6 (59.1;64.0)	84.1 (82.3;85.7)
Yes	20,446	6.4 (5.6;7.5)	22.2 (20.4;24.1)	47.9 (45.4;50.4)	60.1 (57.7;62.5)	87.6 (86.0;89.0)
**Mother’s age (years) (n** **= 37,619)**						
Up to 21	2,627	10.7 (7.7;14.6)	25.6 (21.0;30.8)	59.4 (53.2;65.2)	69.4 (64.4;74.0)	87.2 (83.7;90.0)
22-29	11,565	8.8 (7.0;11.1)	26.8 (24.2;29.5)	55.7 (52.7;58.6)	66.6 (63.8;69.3)	86.5 (84.8;88.1)
30-39	17,472	6.3 (5.3;7.5)	22.6 (20.7;24.6)	46.8 (44.1;49.4)	58.6 (56.1;61.1)	86.3 (84.6;87.9)
40 or over	5,955	5.6 (4.2;7.4)	19.3 (16.3;22.8)	44.3 (39.8;49.0)	55.3 (50.9;59.7)	84.3 (80.5;87.4)

**Figure 1:**
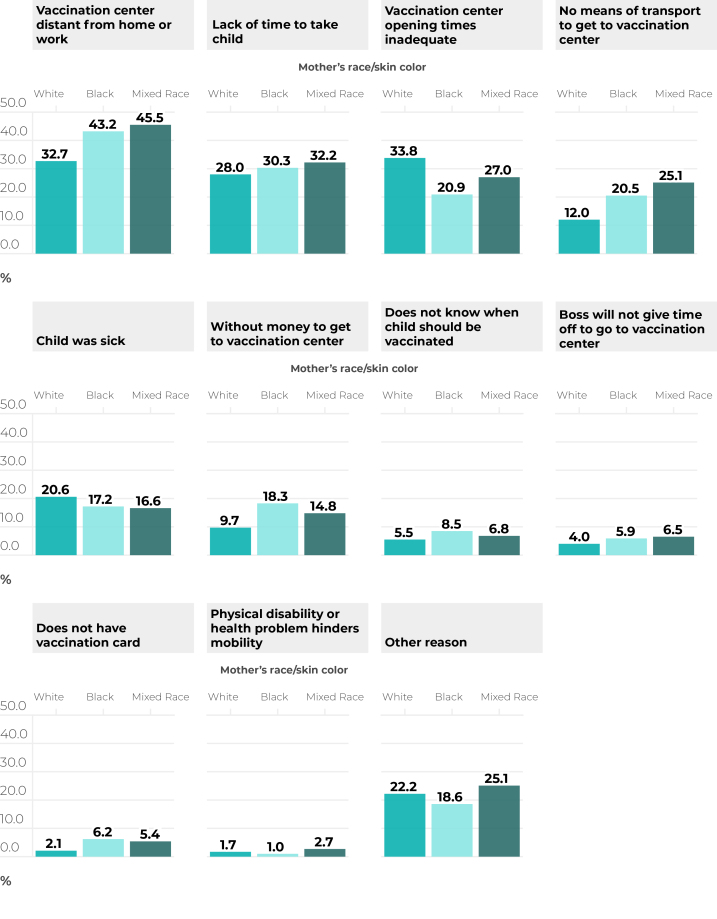
Main reasons given for reporting having faced difficulty in taking child to vaccination center, according to mother’s race/skin color, among live births in Brazil in 2018 and 2018 (n = 37,801)

Figure 1 describes, according to the mothers’ race/skin color, the main difficulties reported in taking children to the vaccination center among respondents who reported some obstacle. The most cited were the distance from home or work to the vaccination center, lack of time to take the child, inadequate opening hours and transportation difficulties in getting to the clinic. The specific measures with the greatest divergence to the detriment people of Black and mixed race/skin color related to traveling to the vaccine center, boss not allowing time off and not having a vaccination card.

**Figure 2:**
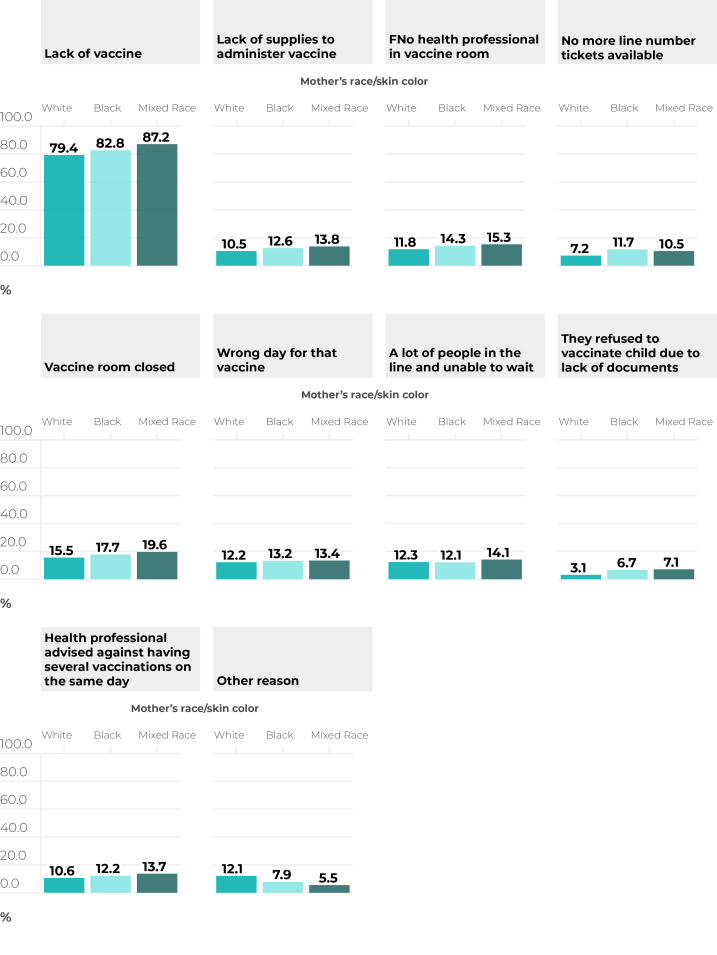
Main reasons given for reporting that child failed to be vaccinated at some time, despite having been taken to the vaccination center, according to mother’s race/skin color, among live births in Brazil in 2018 and 2018 (n = 37,801)

The main reason for not having vaccinated children even after taking them to the vaccination center among those who responded positively to such an occurrence was lack of vaccine (Figure 2). The specific prevalence measures of finding the vaccination room closed, no health professional in the vaccination room, no more line number tickets available and not being vaccinated due to lack of documents were higher in children of Black and mixed race/skin color compared to those of White race/skin color.

Logistic regression analysis indicated greater difficulties in vaccinating children whose mothers were of Black or mixed race/skin color (Table 2). Regarding difficulty in taking the child to the vaccination center, in the crude analysis, we found that this difficulty was 73% (95%CI 30;129) and 75% (95%CI 25;145) higher when the mother was of mixed race or Black, respectively. Furthermore, the odds of not having been vaccinated, despite having been taken to the vaccination center, was 95% (95%CI 69;125) and 97% (95%CI 57;148) higher among those of mixed race and Black race/skin color, respectively. Even after adjusting the analysis for the mother’s schooling, age and work, association remained statistically significant with poorer outcomes for those of Black and mixed race/skin color. In relation to the other variables, only having 13 years of study or more reduced the odds of having difficulty taking the child to the vaccination center.

**Table 2:** Crude and adjusted odds ratios based on logistic regression between exploratory variables and the outcomes of difficulty in taking child to vaccination center and not having child vaccination even after having gone to the health center, among Brazilian children born between 2017 and 2018

	Faced difficulty in taking child to vaccination center		Child not vaccinated, despite having been taken to vaccination center	
	**Crude OR**	**Adjusted OR**	**Crude OR**	**Adjusted OR**
**Race/skin color**				
White	1.00	1.00	1.00	1.00
Mixed race	1.73 (1.30;2.29)	1.54 (1.13;2.10)	1.95 (1.69;2.25)	1.92 (1.66;2.23)
Black	1.75 (1.25;2.45)	1.54 (1.10;2.16)	1.97 (1.57;2.48)	1.96 (1.56;2.45)
**Mother’s schooling (years of study)**				
Up to 8	1.00	1.00	1.00	1.00
9-12	0.91 (0.60;1.38)	0.83 (0.58;1.18)	1.03 (0.79;1.34)	0.98 (0.77;1.24)
13 or over	0.52 (0.38;0.61)	0.61 (0.45;0.84)	1.02 (0.82;1.29)	1.24 (0.99;1.55)
**Mother worked in the last month**				
No	1.00	1.00	1.00	1.00
Yes	0.79 (0.62;1.00)	0.98 (0.80;1.20)	0.84 (0.74;0.96)	0.92 (0.80;1.04)
**Mother’s age (years)**				
Up to 21	1.00	1.00	1.00	1.00
22-29	0.81 (0.54;1.20)	0.86 (0.58;1.26)	1.06 (0.81;1.40)	0.97 (0.73;1.28)
30-39	0.56 (0.38;0.84)	0.71 (0.45;1.11)	0.85 (0.65;1.11)	0.87 (0.66;1.15)
40 or over	0.49 (0.31;0.79)	0.63 (0.37;1.07)	0.70 (0.51;0.96)	0.73 (0.52;1.01)

Regarding incompleteness of vaccination on time, we found that children with mixed race and Black mothers showed, respectively, odds of 1.57 (95%CI 1.36;1.82) and 1.47 (1.18;1.84) times greater delay in vaccination up to 5 months, and 1.62 (95%CI 1.40;1.88) and 1.57 (1.27;1.94) for incomplete vaccination on time in the first year when compared to those with White mothers (Table 3). Even after adjustments, both associations remained statistically significant, with only a small reduction in the magnitude of the values. Mothers with less schooling also had lower odds of completing vaccinations on time in the first 5 months and first year of life, while the odds of poorer outcomes were greater in all cases among mothers up to 21 years of age. In relation to vaccination not being completed on time in the second year of life, no differences were found according to the mothers’ race/skin color.

**Table 3:** Crude and adjusted odds ratios based on logistic regression between exploratory variables and incomplete vaccination on time at 5 months old, at the end of the first year of life and in the second year of life among Brazilian children born between 2017 and 2018

	Delay in scheduled vaccination at up to 5 months old		Incomplete vaccination schedule on time in 1^st^ year of life		Incomplete vaccination schedule on time in 2^nd^ year of life	
	**Crude OR**	**Adjusted OR**	**Crude OR**	**Adjusted OR**	**Crude OR**	**Adjusted OR**
**Race/skin color**						
White	1.00	1.00	1.00		1.00	1.00
Mixed race	1.57 (1.36;1.82)	1.40 (1.21;1.62)	1.62 (1.40;1.88)	1.48 (1.28;1.72)	0.97 (0.79;1.19)	1.01 (0.81;1.26)
Black	1.47 (1.18;1.84)	1.29 (1.03;1.61)	1.57 (1.27;1.94)	1.40 (1.13;1.73)	0.76 (0.58;1.01)	0.79 (0.59;1.06)
**Mother’s schooling (years of study** **)**						
Up to 8	1.00	1.00	1.00	1.00	1.00	1.00
9-12	0.59 (0.45;0.78)	0.55 (0.42;0.71)	0.59 (0.42;0.81)	0.55 (0.40;0.75)	0.89 (0.60;1.31)	0.86 (0.58;1.26)
13 or over	0.40 (0.32;0.51)	0.44 (0.34;0.56)	0.46 (0.35;0.60)	0.50 (0.38;0.66)	1.11 (0.82;1.50)	1.04 (0.75;1.43)
**Mother worked in the last month**						
No	1.00	1.00	1.00	1.00	1.00	1.00
Yes	0.86 (0.76;0.97)	1.04 (0.91;1.19)	0.94 (0.82;1.07)	1.12 (0.98;1.29)	1.33 (1.11;1.59)	1.34 (1.10;1.64)
**Mother’s age (years)**						
Up to 21	1.00	1.00	1.00	1.00	1.00	1.00
22-29	0.86 (0.66;1.12)	0.97 (0.74;1.26)	0.88 (0.69;1.12)	0.96 (0.75;1.24)	0.95 (0.69;1.30)	0.83 (0.59;1.15)
30-39	0.60 (0.47;0.77)	0.75 (0.58;0.97)	0.62 (0.49;0.79)	0.75 (0.58;0.96)	0.93 (0.68;1.23)	0.76 (0.55;1.07)
40 or over	0.54 (0.40;0.75)	0.68 (0.49;0.94)	0.54 (0.41;0.73)	0.64 (0.47;0.87)	0.79 (0.53;1.17)	0.63 (0.41;0.96)

## DISCUSSION

This study identified high frequency of delays in timely vaccination in Brazil during the first 24 months of life and that a high proportion of families face several obstacles to vaccination. Occurrence of these adverse situations is greater among children whose mothers are of Black and mixed race/skin color. Notably, these children are more likely to face difficulties in being taken to the vaccination center, not being vaccinated even when attending the clinic and having incomplete vaccination compared to vaccination schedules in the first 5 and 12 months of life.

Direct comparisons between countries regarding the proportion of children with delayed vaccinations should be made with caution, as the definition of delay and the set of vaccines considered in each study vary greatly. Even so, we found that the proportion of Brazilian children who, in the first year (61.1%) and in the second year of life (86.1%), had not been vaccinated completely on time was high. In the United States, Freeman et al.^11^ found that 58.3% of children had not received all vaccines on time at 19 months of age within a set of seven vaccines. In Quebec, Canada, the percentage of children with up-to-date vaccinations at 12 months of age was 77%,^12^ while in Norway it was 55.3% at 24 months.^13^ In India, it is estimated that 23.1%, 29.3% and 34.8% of children between 10 and 23 months had their BCG, first dose of DPT and measles vaccines delayed, respectively.^14^

Although Brazil has a long history of a successful immunization program, it has faced significant drops in vaccination coverage between 2016 and 2021.^15^ Several factors have been identified for this worrying scenario. One of them is vaccine hesitancy due to fear of side effects from immunobiologicals, lack of trust in health supplies/services, or perception of low risk of disease,^16^ these being feelings that have grown in several countries around the world. Even so, compared to other locations, in Brazil there is still high confidence in vaccines and a high proportion of intention to vaccinate children.^2^

Another important aspect is the complexity of the vaccination schedule.^16^ Inclusion of new immunobiologicals can make it difficult to understand which vaccine to offer one’s child and at what time, in addition to raising concerns about the effect of administering multiple vaccines. If they are not well trained, even health professionals may miss the opportunity for vaccination due to difficulty in understanding the vaccination schedule or handling vaccine hesitancy.

However, despite the relevance of these aspects, our study highlighted material and objective factors that affect the vaccination of children in Brazil. Almost one in four children failed to be vaccinated at some point up to 24 months of age, despite being taken to the vaccination center. Structural and organizational failures of health services have been mentioned. A survey conducted in 2021 with municipal health secretaries in Brazil identified that three out of every four health service managers reported delays in receiving vaccines as a frequent problem.^17^ Another 70.8% indicated that there were problems regarding the quantity of vaccines received and 60.4% stated that frequency of vaccine receipt is irregular. Furthermore, more than half reported that their municipality had an inadequate structure for providing the service to the population, as well as high vaccine room team turnover. Therefore, expanding vaccination coverage must also involve strengthening primary care, expanding and training health teams and qualifying the structure of vaccination centers. All of these aspects involve prioritizing public health and large-scale investments in the SUS. Even so, Brazil has historically lived with low public *per* *capita* investment in health^18^ and, more recently, has lived with changes in primary care that have impacted the composition of health teams and actions in territories that can negatively impact racial inequalities in health.^19^

Difficulties in traveling to the vaccination center, lack of money, restricted center opening hours and lack of time off work were factors cited in the study as obstacles to taking children to be vaccinated. A systematic review conducted by Cavalcanti and Nascimento^20^ found that living within walking distance of a primary health care center increases the chance of vaccination, while a study conducted with users of health services and primary care professionals also identified financial resources needed for travel and health center opening hours as affecting the use of services.^21^

Children born to Black mothers showed higher incidence of delayed vaccination, and their families more frequently reported the presence of barriers to accessing vaccination. An episode of non-vaccination was reported for one in three children of Black mothers, even if the child had been taken to the vaccination center. Similar results have been found in other countries, such as England^22^ and the United States.^23^ However, this result is especially disturbing given the fact that Brazil has a public health system designed on the principles of universality and equity. The structural and organizational failures that cause such losses of vaccination opportunities affect Black and mixed race people more intensely and this finding needs to be considered when designing actions to improve the SUS. This result also needs to spur actions that promote equity beyond average increase in vaccination coverage in Brazil. Previous studies have identified that Black people have greater difficulty accessing health services in Brazil,^24^ have fewer diagnostic tests^25^ and are less likely to see the same doctor in primary care.^26^ This scenario contrasts with the higher prevalence of negative self-rated health in this group,^27^ this being an important proxy for adverse clinical health outcomes. Although the SUS is a vital social policy that promotes equity, its shortcomings tend to have a more intense impact on the most vulnerable segments of the population. In addition to the recent changes in the National Primary Care Policy, health system underfunding and other social policies make the path towards racial equity in Brazil more tortuous.

Furthermore, our study found that material, travel and employment limitations disproportionately affect the Black population in relation to child vaccination. Brazil is marked by pronounced social and economic disparities, where racism manifests itself both in everyday interactions and in institutional structures, permeating different aspects of life. Characteristics such as place of residence, income, urban mobility and autonomy at work differ according to people’s race/skin color.^28-30^ Thus, the inequalities observed in health outcomes are also the product of a society that promotes different opportunities for people according to their education, income and race/skin color. In this sense, it is noteworthy that the racial inequalities observed in this study remained even after adjustment for other socioeconomic characteristics, placing race/skin color at the center of the discussion on equity in vaccination.

This study had limitations. There was significant variation in the proportion of losses between municipalities included in the research and socioeconomic strata used in the sampling process. Furthermore, the analyses were carried out by grouping interviewees from all municipalities, not analyzing possible regional differences in terms of the outcomes investigated. Data collection during the COVID-19 pandemic also impacted response rates. Even so, it is noteworthy that the calculation of post-stratification sample weights took into account differences in responses between population groups and minimized such differences. Strengths of the study include its broad geographic coverage and large sample size, in addition to the methodological rigor involved in collecting vaccination information.

The findings of this study highlight the need for equitable public policies that should aim to remove barriers to vaccination and promote qualification of health services, especially for the Black population. Future studies need to deepen understanding of social and structural determinants that lead to such disparities and investigate intersections with other socioeconomic and demographic dimensions.
