# Supplementary material for: Racial inequalities in child vaccination and barriers to vaccination in Brazil among live births in 2017 and 2018: an analysis of a retrospective cohort of the first two years of life
Source: Epidemiol Serv Saude. 2024 Aug 23;33(spe2):e20231216. doi: 10.1590/S2237-96222024v33e20231216.especial2.en (PMC11346768; doi:10.1590/S2237-96222024v33e20231216.especial2.en)
Supplement: Translation

# Translation: Desigualdades raciais na vacinação infantil e nos obstáculos à vacinação no Brasil entre nascidos vivos em 2017 e 2018: análise de uma coorte retrospectiva dos dois primeiros anos de vida

**DOI:** 10.1590/S2237-96222024v33e20231216.especial2.pt

**Authors:** Antonio Fernando Boing, Alexandra Crispim Boing, Ana Paula França, José Cássio de Moraes, Grupo ICV 2020

**Affiliations:** 1 Universidade Federal de Santa Catarina, Programa de Pós-graduação em Saúde Coletiva, Florianópolis, SC, Brasil; 2 Faculdade de Ciências Médicas Santa Casa de São Paulo, Departamento de Saúde Coletiva, São Paulo, SP, Brasil; 3 Universidade Federal do Espírito Santo, Vitória, ES, Brasil; 4 Universidade Federal do Ceará, Departamento de Saúde Comunitária, Fortaleza, CE, Brasil; 5 Faculdade Ciências Médicas Santa Casa de São Paulo, São Paulo, SP, Brasil; 6 Secretaria de Estado da Saúde do Amapá, Macapá, AP, Brasil; 7 Universidade Federal de Santa Catarina, SC, Brasil; 8 Organização Pan-Americana da Saúde, Brasília, DF, Brasil; 9 Instituto Evandro Chagas, Belém, PA, Brasil; 10 Faculdade de Ciências Médicas Santa Casa de São Paulo, Departamento de Saúde Coletiva, São Paulo, SP, Brasil; 11; 12; 13 Universidade Federal de Mato Grosso, Cuiabá, MT, Brasil; 14 Universidade Federal do Paraná, Curitiba, PR, Brasil; 15 Universidade Federal de Goiás, Goiânia, GO, Brasil; 16 Universidade Federal do Piauí, Teresina, PI, Brasil; 17 Universidade de Pernambuco, Faculdade de Ciências Médicas, Pernambuco, PE, Brasil; 18 Instituto de Saúde Coletiva, Universidade Federal da Bahia, Salvador, BA, Brasil; 19 Secretaria de Estado da Saúde de Alagoas, Maceió, AL, Brasil; 20 Universidade Federal do Acre, Rio Branco, AC, Brasil; 21 Universidade Federal do Maranhão, Departamento de Saúde Pública, São Luís, MA, Brasil; 22 Universidade Federal de Sergipe, Aracaju, SE, Brasil; 23 Secretaria Municipal de Saúde, Boa Vista, RR, Brasil; 24 Fundação Oswaldo Cruz, Mato Grosso do Sul, Campo Grande, MS, Brasil; 25 Fundação Oswaldo Cruz, Escola Nacional de Saúde Pública Sergio Arouca, Rio de Janeiro, RJ, Brasil; 26 Universidade Federal do Rio Grande do Sul, Porto Alegre, RS, Brasil; 27 Fundação Oswaldo Cruz, Instituto de Pesquisa René Rachou, Belo Horizonte, MG, Brasil; 28 Secretaria de Desenvolvimento Ambiental de Rondônia, Porto Velho, RO, Brasil; 29 Universidade de Brasília, Brasília, DF, Brasil

**Keywords:** Cobertura Vacinal, Criança, Iniquidades em Saúde, Inquéritos Epidemiológicos, Brasil

## Abstract

**Objetivo:**

Descrever a completude vacinal em tempo oportuno nos primeiros 24 meses de vida no Brasil e os obstáculos para vacinação, testando-se associações com raça/cor da pele materna.

**Métodos:**

Fez-se coleta de informações sobre os nascidos em 2017 e 2018, constantes no Inquérito Nacional de Cobertura Vacinal. Foram calculados prevalência e intervalos de confiança de 95% de obstáculos à vacinação e completude vacinal em tempo oportuno aos 5 meses, primeiro e segundo ano, segundo raça/cor da pele materna. Empregou-se regressão logística para análise de associações.

**Resultados:**

Analisaram-se dados de 37.801 crianças. Do total, 7,2% (IC_95_% 6,3;8,2) dos responsáveis enfrentaram dificuldades para levar seus filhos para vacinação e 23,4% (IC_95_% 21,7;25,1) das crianças não foram vacinadas, mesmo sendo levadas. Essas proporções foram 75% (IC_95_% 1,25;2,45) e 97% (IC_95_% 1,57;2,48) mais elevadas, respectivamente, entre pretas; e 49,9% (IC_95_% 47,8;51,9) e 61,1% (IC_95_% 59,2;63,0) das crianças tiveram atraso em alguma vacina até os 5 meses e o primeiro ano, respectivamente. Tais valores foram maiores entre pardas/pretas.

**Conclusão:**

Há desigualdades raciais nos obstáculos enfrentados e na vacinação no Brasil.

## Contribuições do estudo

**Principais resultados:**

Observaram-se marcantes desigualdades raciais nos obstáculos à vacinação de crianças menores de 24 meses no Brasil e à vacinação em tempo oportuno aos 5 meses e no primeiro ano de vida.

**Implicações para os serviços:**

As desigualdades raciais na ocorrência de falhas nos serviços de saúde para vacinação, nas restrições objetivas das famílias para levar a criança ao posto de vacinação e na incompletude vacinal em tempo oportuno precisam ser enfrentadas pelo SUS.

**Perspectivas:**

Políticas públicas equânimes para enfrentamento às barreiras à vacinação e qualificação dos serviços de saúde precisam ser implementadas. Estudos devem aprofundar a compreensão dos determinantes estruturais que levam às disparidades raciais.

## INTRODUÇÃO

A imunização desempenha papel fundamental na prevenção de doenças infecciosas e na redução da mortalidade infantil, mas sua distribuição efetiva é desigual tanto entre países quanto dentro deles.^1^ A Organização Mundial da Saúde (OMS) estima que, na década de 2010, mais de 20 milhões de bebês não completavam o esquema básico de vacinação no mundo a cada ano e, desses, mais de 13 milhões não recebiam qualquer vacina por meio dos programas de imunização.^2^

A OMS também estabeleceu como um de seus objetivos fornecer serviços de vacinação acessíveis a todas as pessoas em todos os lugares até 2030, com o intuito de salvar mais de 50 milhões de vidas.^2^ No entanto, um dos desafios cruciais que se apresentam é superar as disparidades existentes nos países. Entre as múltiplas desigualdades que desafiam o pleno desenvolvimento humano estão aquelas relacionadas às características étnico-raciais. Assim, pesquisas que lancem luz sobre elas são essenciais para aprimorar políticas públicas em busca da equidade,^3^ sendo as disparidades raciais na vacinação infantil influenciadas pela cor da pele e por uma miríade de fatores sociais, econômicos e de saúde. 

Uma investigação que abrangeu 64 países de baixa e média renda observou disparidades significativas na imunização infantil relacionadas à etnia em mais da metade desses países.^4^ Diversos estudos apontam que negros enfrentam barreiras significativas no acesso a vacinas, levando a uma menor taxa de imunização quando comparadas a outros grupos raciais.^5,6^ Análises do Centros de Controle e Prevenção de Doenças (Center for Disease Control and Prevention – CDC) indicam que nos Estados Unidos a cobertura da vacina contra covid-19 foi menor entre crianças afro-americanas de 5 a 11 anos.^7^ Outros estudos também mostram piores coberturas entre pretos e/ou grupos minoritários^8^ tanto na vacinação quanto na intenção de vacinar.^9^

Essa desigualdade pode resultar em maiores taxas de doenças preveníveis por vacinação e contribuir para perpetuar as disparidades na saúde.^1,2^ O Brasil é marcado por profundas desigualdades raciais, sociais e econômicas, com reflexos profundos nos níveis de saúde de sua população. Assim, ampliar o conhecimento sobre diferenças de raça/cor da pele no acesso à vacinação e na completude vacinal em tempo oportuno entre crianças é necessário para avaliar e qualificar políticas públicas, em especial diante de um sistema de saúde com o princípio da equidade.

O objetivo do estudo foi descrever a magnitude da completude vacinal em tempo oportuno ao longo dos primeiros 24 meses de vida no Brasil e os obstáculos enfrentados para vacinação, testando-se suas associações com a raça/cor da pele das mães.

## MÉTODOS

### Origem dos dados

Analisaram-se os dados do Inquérito Nacional de Cobertura Vacinal (INCV), uma coorte retrospectiva realizada no Brasil entre setembro de 2020 e março de 2022. Incluíram-se no estudo todas as crianças nascidas em 2017 e 2018 e foram coletados os dados das vacinas recebidas nos primeiros 24 meses de vida. Por meio da análise das cadernetas vacinais das crianças, verificou-se a evolução de suas exposições às vacinas recomendadas em cada idade ao longo desses dois anos iniciais de vida. Foram analisadas as coberturas vacinais no quinto, no décimo segundo e no vigésimo quarto mês de vida. O estudo foi realizado em todas as 26 capitais do Brasil, no Distrito Federal (DF) e em outras 12 cidades com mais de 100 mil habitantes distribuídas em todas as regiões do país, à exceção da região Norte, em áreas não metropolitanas.

### Cálculo da amostra

O cálculo do tamanho amostral seguiu como parâmetros uma prevalência estimada de cobertura vacinal de 70%, erro de estimativa igual a 5%, intervalo de confiança de 95% e efeito do desenho pelo uso de conglomerados igual a 1,4, obtendo-se amostra de 452 crianças por inquérito. Em cada município, realizaram-se de um a quatro inquéritos, a depender do número de nascidos vivos observados em 2017 e 2018, totalizando 89 inquéritos. Foram considerados quatro estratos socioeconômicos dos municípios segundo características de renda e escolaridade dos setores censitários. Mais detalhes do procedimento amostral estão apresentados em publicação prévia.^10^

### Coleta dos dados

Os endereços das crianças foram obtidos no Sistema de Informações sobre Nascidos Vivos (Sinasc), que congrega informações de todos os nascimentos ocorridos no Brasil. Aos entrevistados foi aplicado, por meio de dispositivo eletrônico, questionário estruturado fechado com questões sobre o perfil socioeconômico do domicílio e sociodemográfico da mãe e do responsável pela criança, caso não fosse a mãe. Também foram solicitadas informações sobre percepções quanto às vacinas e barreiras para a vacinação. Adicionalmente, foi solicitada a caderneta de vacinação da criança para registro fotográfico. Todos os dados observados nas fotos foram digitados por profissionais com conhecimento do calendário nacional de imunização, de modo a serem registradas as vacinas aplicadas e as respectivas datas de aplicação. A coordenação nacional do estudo realizou análise de consistência dos dados.

### Desfechos

Inicialmente analisaram-se dois desfechos relacionados a dificuldades objetivas de se vacinar a criança. No primeiro, indagou-se o respondente se já houve alguma dificuldade para levar a criança ao posto de vacinação. No segundo, a pergunta foi se alguma vez a criança deixou de ser vacinada, mesmo tendo sido levada ao posto de vacinação. As opções de resposta, em ambos os casos, foram “sim”, “não” e “não sabe/não quis informar”. Para as pessoas que responderam afirmativamente à primeira pergunta, seguiu-se o questionamento se a dificuldade se deu porque (1) não se tinha a caderneta de vacinação, (2) havia falta de tempo para levar a criança, (3) o horário de funcionamento do posto era inadequado, (4) o posto ficava longe de casa ou do trabalho, (5) o patrão não liberava para ir ao posto de vacinação, (6) não tinha meios de transporte para ir ao posto de vacinação, (7) não tinha dinheiro para ir ao posto, (8) tinha deficiência física ou problema de saúde que dificultava a locomoção do responsável pela criança, (9) não sabia quando a criança devia tomar as vacinas, (10) a criança estava doente, (11) outro motivo.

De forma semelhante, foram perguntados os motivos àqueles que relataram episódios de não vacinação da criança mesmo levando-a ao posto de vacinação: (1) faltou vacina, (2) faltou material para aplicar a vacina, (3) faltou profissional na sala de vacina, (4) acabou a senha, (5) a sala de vacina estava fechada, (6) não era dia da vacina desejada, (7) tinha muita gente na fila e não pôde esperar, (8) não vacinaram a criança porque não tinha documento (como comprovante de residência, cartão SUS, ou caderneta de vacinação), (9) o profissional de saúde não recomendou aplicar várias vacinas no mesmo dia e mandou voltar outro dia, (10) outro motivo. A cada uma dessas indagações a pessoa respondia “sim”, “não” ou “não sabe/não deseja responder”.

Em relação à vacinação, foram analisados três desfechos. Cada um considerou um recorte de tempo ao longo dos primeiros dois anos de vida da criança, conforme explicitado a seguir:

Atraso ou não acesso às vacinas que deveriam ser tomadas nos primeiros cinco meses de vida em tempo oportuno: atraso na vacinação das vacinas BCG e/ou hepatite B e/ou pentavalente (1ª ou 2ª dose) e/ou VIP (1ª ou 2ª dose) e/ou rotavírus (1ª ou 2ª dose) e/ou pneumocócica (1ª ou 2ª dose) e/ou meningo C (1ª ou 2ª dose).

Esquema vacinal incompleto no tempo oportuno no 1º ano de vida: bacilo de Calmette-Guérin (BCG), hepatite B, três doses de vacina pentavalente (tríplice bacteriana + *Haemophilus influenzae* tipo B + hepatite B) e de vacina inativada de poliovírus (VIP), duas doses de vacina contra rotavírus, duas doses de vacina contra meningococo C e pneumococo. 

Esquema vacinal incompleto no tempo oportuno no 2º ano de vida: duas doses de tríplice viral (sarampo, rubéola e parotidite), uma dose de hepatite A, varicela e vacina oral de poliovírus atenuados (VOP); e uma dose de reforço com tríplice bacteriana (difteria, tétano e pertússis), meningococo C e pneumococo.

A definição se as doses foram oportunas e se houve atraso ou não se deu de acordo com o momento em que foram aplicadas, assumindo-se como base a data de nascimento e considerando-se o intervalo entre as doses. A sistematização dos intervalos considerados em cada vacina e dose pode ser encontrada em Barata et al.^10^

### Covariáveis

A variável de principal interesse foi a raça/cor da pele autorreferida da mãe da criança. As opções de resposta seguiram o utilizado pelo Instituto Brasileiro de Geografia e Estatística (IBGE) nas pesquisas conduzidas no Brasil: branca, parda, preta, indígena e amarela. As pessoas que referiram raça/cor da pele indígena ou amarela não foram analisadas no presente estudo em função do reduzido número de observações. Adicionalmente, como variáveis de ajuste, foram incluídas no estudo a escolaridade materna (até 8 anos de estudo, 9 a 12 e 13 ou mais), o trabalho ou estágio materno durante pelo menos uma hora por semana, no último mês, em alguma atividade remunerada em dinheiro (sim ou não), e a idade materna (até 21 anos de idade, 22 a 29 anos, 30 a 39 anos e 40 ou mais anos).

### Análise dos dados

Inicialmente foi descrita a composição da amostra segundo as covariáveis e estimadas as prevalências, com respectivos intervalos de confiança de 95% (IC_95_%), dos cinco desfechos para toda a amostra e segundo cada categoria das variáveis incluídas no estudo. Em seguida, foram calculadas as frequências relativas de cada uma das 11 dificuldades incluídas no estudo para levar a criança ao posto de vacinação e de cada uma das dez razões explicativas para a não vacinação da criança, mesmo levando-a ao posto de vacinação. Por fim, por meio de regressão logística, foram estimadas razões de chances (*odds ratio*) brutas e ajustadas, com respectivos IC_95_%, entre os desfechos e as covariáveis. Todas as análises consideraram os pesos amostrais e o delineamento do estudo, tendo sido realizadas no programa Stata 15.1. O INCV foi aprovado pelo Comitê de Ética em Pesquisa com Seres Humanos da Irmandade da Santa Casa de São Paulo sob parecer nº 4.380.019.

## RESULTADOS

Foram analisados os dados de 37.801 crianças, havendo 6% de perda no estudo. A maior parte dos respondentes referiu raça/cor da pele parda (46,4%), tinha mais de 12 anos de estudo (76,1%), estava na faixa etária de 30 a 39 anos (46,5%) e havia trabalhado no mês anterior à entrevista (55,5%). Verificou-se que 7,2% enfrentaram dificuldades para levar seus filhos ao posto de vacinação e 23,4% das crianças não foram vacinadas mesmo após serem levadas ao posto (Tabela 1). Além disso, 49,9% das crianças tiveram atraso em alguma vacina do calendário até os 5 meses de vida. No primeiro e no segundo ano de vida, 61,1% e 86,1% das crianças, respectivamente, apresentaram esquema vacinal incompleto no tempo oportuno. Observou-se que a prevalência de dificuldades para vacinar, atraso na vacinação e esquema vacinal incompleto no primeiro ano de vida foram mais elevados entre as mães de raça/cor da pele parda e preta em relação às brancas. Em geral, também foram verificados piores indicadores entre aquelas com menor escolaridade e entre mães com menos de 21 anos de idade.

**Tabela 1:** Características maternas da amostra e prevalência de desfechos analisados em relação à cobertura vacinal em tempo oportuno e dificuldades para vacinar as crianças nascidas no Brasil em 2017 e 2018

	Amostra	Enfrentou alguma dificuldade para levar a criança ao posto de vacinação	A criança deixou de ser vacinada, apesar de ter sido levada ao posto de vacinação	Atrasou alguma vacina do calendário até 5 meses de vida	Esquema vacinal incompleto no tempo oportuno no 1º ano de vida	Esquema vacinal incompleto no tempo oportuno no 2º ano de vida
	n	% (IC95%)				
Todos	37.801	7,2 (6,3;8,2)	23,4 (21,7;25,1)	49,9 (47,8;51,9)	61,1 (59,2;63,0)	86,1 (84,9;87,2)
Raça/cor da pele da mãe (n = 36.341)						
Branca	15.227	5,1 (4,1;6,5)	17,3 (15,5;19,2)	43,6 (40,8;46,4)	54,6 (51,8;57,4)	86,5 (84,5;88,4)
Parda	16.859	8,6 (7,5;9,8)	28,9 (26,8;31,2)	53,2 (48,2;58,2)	66,2 (63,9;68,4)	86,2 (84,6;87,6)
Preta	4.255	8,7 (6,9;10,9)	29,2 (25,2;33,5)	54,9 (52,5;57,5)	65,4 (60,9;69,7)	83,1 (79,8;85,9)
Escolaridade da mãe (anos de estudo) (n = 37.748)						
Até 8	3.280	11,2 (8,6;14,3)	23,2 (19,5;27,4)	68,0 (62,8;72,8)	75,4 (70,2;80,0)	85,3 (81,3;88,6)
9-12	5.494	10,2 (7,6;13,7)	23,8 (20,2;27,8)	55,8 (51,0;60,5)	64,3 (59,5;68,8)	83,8 (79,7;87,2)
13 ou mais	27.974	6,1 (5,3;7,0)	25,6 (23,4;29,9)	49,5 (46,6;52,3)	60,7 (58,1;63,2)	84,1 (82,3;85,7)
A mãe trabalhou no último mês (n = 36.866)						
Não	16.420	8,0 (6,7;9,7)	25,3 (23,0;27,7)	51,7 (49,1;54,3)	61,6 (59,1;64,0)	84,1 (82,3;85,7)
Sim	20.446	6,4 (5,6;7,5)	22,2 (20,4;24,1)	47,9 (45,4;50,4)	60,1 (57,7;62,5)	87,6 (86,0;89,0)
Idade da mãe (anos) (n = 37.619)						
Até 21	2.627	10,7 (7,7;14,6)	25,6 (21,0;30,8)	59,4 (53,2;65,2)	69,4 (64,4;74,0)	87,2 (83,7;90,0)
22-29	11.565	8,8 (7,0;11,1)	26,8 (24,2;29,5)	55,7 (52,7;58,6)	66,6 (63,8;69,3)	86,5 (84,8;88,1)
30-39	17.472	6,3 (5,3;7,5)	22,6 (20,7;24,6)	46,8 (44,1;49,4)	58,6 (56,1;61,1)	86,3 (84,6;87,9)
40 ou mais	5.955	5,6 (4,2;7,4)	19,3 (16,3;22,8)	44,3 (39,8;49,0)	55,3 (50,9;59,7)	84,3 (80,5;87,4)

A Figura 1 descreve, segundo raça/cor da pele das mães, as principais dificuldades referidas para levar a criança ao posto de vacinação entre respondentes que relataram algum obstáculo. Os mais citados foram a distância do posto de vacinação em relação à casa ou ao trabalho, a falta de tempo para levar a criança, o horário inadequado de funcionamento e dificuldades de transporte para ir ao posto. As medidas pontuais com maior divergência em desfavor dos pardos e pretos foram relacionadas ao deslocamento até o posto, não liberação do patrão e não dispor de caderneta de vacinação.

**Figura 1:** Principais dificuldades relatadas para levar a criança ao posto de vacinação, segundo a raça/cor da pele da mãe entre nascidos vivos no Brasil em 2017 e 2018 (n = 37.801)

Já o principal motivo para não ter vacinado a criança mesmo levando-a ao posto de vacinação, entre aqueles que responderam positivamente a tal ocorrência, foi a falta de vacina (Figura 2). As medidas pontuais de prevalência de encontrar a sala de vacina fechada, faltar profissional da sala de vacina, ter acabado a senha e não vacinação por falta de documento foram mais elevadas em filhos de pardas e pretas em relação a brancas.

**Figura 2:** Principais motivos relatados para a criança não ter sido vacinada alguma vez, apesar de ter sido levada ao posto de vacinação, segundo a raça/cor da pele da mãe, entre crianças nascidas no Brasil em 2017 e 2018 (n = 37.801)

A análise de regressão logística indicou maiores dificuldades para vacinação de crianças cujas mães eram pretas ou pardas (Tabela 2). Quanto à dificuldade para levar a criança ao posto de vacinação, na análise bruta, verificou-se que ela foi 73% (IC_95_% 30;129) e 75% (IC_95_% 25;145) maior quando a mãe era parda ou preta, respectivamente. Além disso, a chance de não ter sido vacinada, apesar de ter sido levada ao posto de vacinação, foi 95% (IC_95_% 69;125) e 97% (IC_95_% 57;148) maior entre pardas e pretas, respectivamente. Mesmo após se ajustar a análise pela escolaridade, idade e trabalho da mãe, a associação se manteve estatisticamente significativa com piores desfechos para as pardas e pretas. Em relação às demais variáveis, apenas ter 13 anos de estudo ou mais diminuiu a chance de se enfrentar dificuldade para levar a criança ao posto de vacinação.

**Tabela 2:** Valores de odds ratio brutas e ajustadas a partir da regressão logística entre variáveis exploratórias e as dificuldades para levar a criança ao posto de vacinação e ela não ser vacinada mesmo após ida à unidade de saúde, entre crianças nascidas no Brasil em 2017 e 2018

	Enfrentou alguma dificuldade para levar a criança ao posto de vacinação		A criança deixou de ser vacinada, apesar de ter sido levada ao posto de vacinação	
	OR bruta	OR ajustada	OR bruta	OR ajustada
Raça/cor da pele				
Branca	1,00	1,00	1,00	1,00
Parda	1,73 (1,30;2,29)	1,54 (1,13;2,10)	1,95 (1,69;2,25)	1,92 (1,66;2,23)
Preta	1,75 (1,25;2,45)	1,54 (1,10;2,16)	1,97 (1,57;2,48)	1,96 (1,56;2,45)
Escolaridade da mãe (anos de estudo)				
Até 8	1,00	1,00	1,00	1,00
9-12	0,91 (0,60;1,38)	0,83 (0,58;1,18)	1,03 (0,79;1,34)	0,98 (0,77;1,24)
13 ou mais	0,52 (0,38;0,61)	0,61 (0,45;0,84)	1,02 (0,82;1,29)	1,24 (0,99;1,55)
A mãe trabalhou no último mês				
Não	1,00	1,00	1,00	1,00
Sim	0,79 (0,62;1,00)	0,98 (0,80;1,20)	0,84 (0,74;0,96)	0,92 (0,80;1,04)
Idade da mãe (anos)				
Até 21	1,00	1,00	1,00	1,00
22-29	0,81 (0,54;1,20)	0,86 (0,58;1,26)	1,06 (0,81;1,40)	0,97 (0,73;1,28)
30-39	0,56 (0,38;0,84)	0,71 (0,45;1,11)	0,85 (0,65;1,11)	0,87 (0,66;1,15)
40 ou mais	0,49 (0,31;0,79)	0,63 (0,37;1,07)	0,70 (0,51;0,96)	0,73 (0,52;1,01)

Em relação à incompletude vacinal em tempo oportuno, observou-se que filhos de mães pardas e pretas apresentaram, respectivamente, chances 1,57 (IC_95_% 1,36;1,82) e 1,47 (1,18;1,84) vezes maiores de atraso na vacinação até os 5 meses, e de 1,62 (IC_95_% 1,40;1,88) e 1,57 (1,27;1,94) para esquema vacinal incompleto em tempo oportuno no primeiro ano, quando comparadas aos de mães brancas (Tabela 3). Mesmo após ajustes, ambas as associações permaneceram estatisticamente significativas, havendo apenas pequena redução na magnitude dos valores. As mães com menor escolaridade também apresentaram menor chance de completude vacinal em tempo oportuno nos primeiros 5 meses e no primeiro ano de vida, enquanto as chances de piores desfechos foram maiores, em todos os casos, entre mães com até 21 anos de idade. Já em relação à incompletude vacinal em tempo oportuno no segundo ano de vida, não foram observadas diferenças segundo raça/cor da pele das mães.

**Tabela 3:** Valores de odds ratio brutas e ajustadas a partir da regressão logística entre variáveis exploratórias e incompletude vacinal em tempo oportuno aos 5 meses de idade, ao final do primeiro ano de vida e no segundo ano de vida, entre crianças nascidas no Brasil em 2017 e 2018

	Atrasou alguma vacina do calendário até 5 meses de vida		Esquema vacinal incompleto no tempo oportuno no 1º ano de vida		Esquema vacinal incompleto no tempo oportuno no 2º ano de vida	
	OR bruta	OR ajustada	OR bruta	OR ajustada	OR bruta	OR ajustada
Raça/cor da pele						
Branca	1,00	1,00	1,00		1,00	1,00
Parda	1,57 (1,36;1,82)	1,40 (1,21;1,62)	1,62 (1,40;1,88)	1,48 (1,28;1,72)	0,97 (0,79;1,19)	1,01 (0,81;1,26)
Preta	1,47 (1,18;1,84)	1,29 (1,03;1,61)	1,57 (1,27;1,94)	1,40 (1,13;1,73)	0,76 (0,58;1,01)	0,79 (0,59;1,06)
Escolaridade da mãe (anos de estudo)						
Até 8	1,00	1,00	1,00	1,00	1,00	1,00
9-12	0,59 (0,45;0,78)	0,55 (0,42;0,71)	0,59 (0,42;0,81)	0,55 (0,40;0,75)	0,89 (0,60;1,31)	0,86 (0,58;1,26)
13 ou mais	0,40 (0,32;0,51)	0,44 (0,34;0,56)	0,46 (0,35;0,60)	0,50 (0,38;0,66)	1,11 (0,82;1,50)	1,04 (0,75;1,43)
A mãe trabalhou no último mês						
Não	1,00	1,00	1,00	1,00	1,00	1,00
Sim	0,86 (0,76;0,97)	1,04 (0,91;1,19)	0,94 (0,82;1,07)	1,12 (0,98;1,29)	1,33 (1,11;1,59)	1,34 (1,10;1,64)
Idade da mãe (anos)						
Até 21	1,00	1,00	1,00	1,00	1,00	1,00
22-29	0,86 (0,66;1,12)	0,97 (0,74;1,26)	0,88 (0,69;1,12)	0,96 (0,75;1,24)	0,95 (0,69;1,30)	0,83 (0,59;1,15)
30-39	0,60 (0,47;0,77)	0,75 (0,58;0,97)	0,62 (0,49;0,79)	0,75 (0,58;0,96)	0,93 (0,68;1,23)	0,76 (0,55;1,07)
40 ou mais	0,54 (0,40;0,75)	0,68 (0,49;0,94)	0,54 (0,41;0,73)	0,64 (0,47;0,87)	0,79 (0,53;1,17)	0,63 (0,41;0,96)

## DISCUSSÃO

O presente estudo identificou no Brasil alta frequência de atraso na vacinação oportuna durante os primeiros 24 meses de vida e que elevada proporção de famílias enfrenta diversos obstáculos para a vacinação. A ocorrência dessas situações adversas é maior entre crianças cujas mães são de raça/cor da pele parda e preta. Notadamente, essas crianças têm maior chance de enfrentar dificuldades para serem levadas ao posto de vacinação, de não serem vacinadas mesmo após chegarem ao posto e de possuírem esquemas vacinais incompletos nos primeiros 5 e 12 meses de vida.

Comparações diretas entre países na proporção de crianças com atraso vacinal devem ser realizadas com cautela, pois a definição de atraso e o conjunto de vacinas consideradas em cada estudo apresentam grande variação. Ainda assim, observou-se que a proporção de crianças brasileiras que, no primeiro ano (61,1%) e no segundo ano de vida (86,1%), apresentavam esquema vacinal incompleto em tempo oportuno foi elevada. Nos Estados Unidos, Freeman et al.^11^ observaram que 58,3% das crianças não tinham recebido todas as vacinas em tempo oportuno aos 19 meses de vida, dentro de um conjunto de sete vacinas. Já em Quebec, Canadá, o percentual de crianças com vacinas em dia aos 12 meses de vida foi de 77%,^12^ enquanto na Noruega foi de 55,3% aos 24 meses.^13^ Na Índia, estima-se que 23,1%, 29,3% e 34,8% das crianças entre 10 e 23 meses receberam com atraso as vacinas para BCG, primeira dose de dTpa e sarampo, respectivamente.^14^

O Brasil, apesar de ter longa história de um bem-sucedido programa de vacinação, enfrentou expressivas quedas na cobertura vacinal entre 2016 e 2021.^15^ Diversos fatores têm sido identificados para esse preocupante cenário. Um deles é a hesitação vacinal por medo de efeitos colaterais dos imunobiológicos, pela falta de confiança nos insumos/serviços de saúde, ou percepção de baixo risco das doenças,^16^ sentimentos que têm crescido em diversos países do mundo. Ainda assim, comparando-se a outras localidades, no Brasil ainda há alta confiança nas vacinas e elevada proporção de intenção de vacinar as crianças.^2^

Outro aspecto importante é a complexidade do calendário vacinal.^16^ A inclusão de novos imunobiológicos pode dificultar o entendimento sobre qual vacina oferecer ao filho e em qual momento, além de poder gerar preocupações sobre o efeito da aplicação de múltiplas vacinas. Caso não estejam bem capacitados, inclusive os profissionais de saúde podem perder a oportunidade de vacinação por dificuldade no entendimento do calendário de vacinação ou no manejo de hesitação vacinal.

No entanto, apesar da relevância desses aspectos, destacaram-se no presente estudo, fatores materiais e objetivos que afetam a vacinação de crianças no Brasil. Quase uma em cada quatro crianças deixou de ser vacinada, em algum momento, até os 24 meses de vida, apesar de ter sido levada ao posto de vacinação. Falhas estruturais e organizacionais dos serviços de saúde foram mencionadas. Pesquisa em 2021 com secretários municipais de saúde do Brasil identificou que três em cada quatro gestores relataram atraso no recebimento de vacina como um problema frequente.^17^ Outros 70,8% indicaram haver problemas quanto à quantidade de vacinas recebidas e 60,4% afirmaram que a periodicidade no recebimento é irregular. Além disso, mais da metade relatou haver no município estrutura inadequada para atender a população e alta rotatividade da equipe da sala de vacina. Assim, a ampliação da cobertura vacinal deve passar também pelo fortalecimento da atenção primária, ampliação e treinamento das equipes de saúde e qualificação da estrutura dos postos de vacinação. Todos esses aspectos envolvem priorização da saúde pública e fortes investimentos no Sistema Único de Saúde (SUS). Ainda assim, o Brasil tem convivido historicamente com baixo investimento público *per capita* em saúde^18^ e, mais recentemente, com mudanças na atenção primária, que impactaram na composição das equipes de saúde e nas ações nos territórios que podem impactar negativamente nas desigualdades raciais em saúde.^19^

Dificuldades de deslocamento até o posto de vacinação, falta de dinheiro, horário restrito de funcionamento do posto e não liberação do trabalho foram fatores citados no estudo como obstáculos para levar a criança para vacinar. Revisão sistemática conduzida por Cavalcanti e Nascimento^20^ verificou que residir a curta distância da unidade básica de saúde (UBS) aumenta a chance de vacinação, enquanto estudo com usuários dos serviços de saúde e profissionais da atenção primária também identificou o recurso financeiro para deslocamento e o horário de funcionamento das unidades como moduladores do uso dos serviços.^21^

Crianças nascidas de mães negras demonstraram ocorrência mais elevada de atrasos na vacinação, e suas famílias relataram com maior frequência a presença de barreiras de acesso à vacinação. Para uma em cada três crianças de mães pretas houve relato de algum episódio de não vacinação, mesmo levando-se a criança ao posto de vacinação. Resultados semelhantes foram encontrados também em outros países, como Inglaterra^22^ e Estados Unidos.^23^ No entanto, esse resultado é especialmente perturbador pelo fato de o Brasil ter um sistema público de saúde concebido nos princípios de universalidade e equidade. As falhas estruturais e organizacionais que ocasionam tais perdas de oportunidade de vacinação afetam com mais intensidade as pessoas pretas e pardas, e tal constatação precisa ser considerada quando desenhadas ações de aperfeiçoamento do SUS. Esse resultado precisa também motivar ações que promovam equidade para além do aumento médio da cobertura vacinal no Brasil. Estudos anteriores já identificaram que pessoas negras têm maior dificuldade de acesso aos serviços de saúde no Brasil,^24^ menor realização de testes diagnósticos^25^ e menor probabilidade de se consultarem com o mesmo médico na atenção primária.^26^ Tal cenário contrasta com a maior prevalência nesse grupo de autoavaliação negativa de saúde,^27^ uma importante *proxy* de desfechos clínicos adversos em saúde. Ainda que o SUS seja vital na promoção de política social de equidade, suas deficiências tendem a impactar de maneira mais intensa os segmentos mais vulnerabilizados da população. Além das recentes mudanças na Política Nacional de Atenção Básica, o subfinanciamento do sistema de saúde e de outras políticas sociais torna mais tortuoso o caminho em busca da equidade racial no país.

Adicionalmente, o presente estudo verificou que limitações materiais, de deslocamento e de emprego afetam desproporcionalmente a população negra em relação à vacinação das crianças. O Brasil é marcado por acentuadas disparidades sociais e econômicas, manifestando-se o racismo tanto em interações cotidianas quanto em estruturas institucionais, permeando diversos aspectos da vida. Características como local de moradia, renda, mobilidade urbana e autonomia no trabalho são diferenciados de acordo com a raça/cor da pele das pessoas.^28-30^ Assim, as desigualdades observadas nos desfechos em saúde são também produto de uma sociedade que promove oportunidades diferentes às pessoas de acordo com sua escolaridade, renda e raça/cor da pele. Nesse sentido, inclusive, destaca-se que as desigualdades raciais observadas no presente estudo se mantiveram mesmo após ajuste por outras características socioeconômicas, colocando a raça/cor da pele no centro da discussão sobre equidade na vacinação.

O presente estudo teve limitações. Houve expressiva variação na proporção de perdas entre municípios incluídos na pesquisa e estratos socioeconômicos usados no processo amostral. Além disso, as análises foram realizadas agrupando-se os entrevistados de todos os municípios, não se analisando possíveis diferenças regionais quanto aos desfechos investigados. A coleta de dados durante a pandemia pela covid-19 também impactou as taxas de resposta. Ainda assim, destaca-se que o cálculo de pesos amostrais pós-estratificação contemplou diferenças nas respostas entre grupos populacionais e minimizou tais diferenças. Como pontos fortes, destacam-se a ampla cobertura geográfica e o elevado tamanho amostral, além do rigor metodológico envolvido na coleta das informações vacinais.

Os achados do presente estudo ressaltam a necessidade de políticas públicas equânimes, que devem visar a remoção de barreiras à vacinação e qualificação dos serviços de saúde, em especial quanto à população negra. Estudos futuros devem aprofundar a compreensão dos determinantes sociais e estruturais que levam a tais disparidades e investigar intersecções com outras dimensões socioeconômicas e demográficas.
